# Tackling a global threat: a clinical scenario-based framework for preventing and managing *Candidozyma auris* infections

**DOI:** 10.3389/fcimb.2026.1796121

**Published:** 2026-03-10

**Authors:** Jinghua Ji, Wenting Xu, Yi Chen

**Affiliations:** 1 Department of Infection Control, Zhangjiagang Traditional Chinese Medicine Hospital Affiliated to Nanjing University of Chinese Medicine, Zhangjiagang, Jiangsu, China; 2 Department of Reproduction, Zhangjiagang Traditional Chinese Medicine Hospital Affiliated to Nanjing University of Chinese Medicine, Zhangjiagang, Jiangsu, China; 3 Department of Gynecology, Kunshan Hospital of Traditional Chinese Medicine Affiliated to Nanjing University of Chinese Medicine, Kunshan, Jiangsu, China

**Keywords:** antifungal resistance, *Candidozyma auris*, clinical scenario, control bundle, fungemia, healthcare-associated infection, infection prevention and control, invasive candidiasis

## Abstract

Candidozyma auris (formerly Candida auris) has emerged as a formidable global health threat, characterized by its multidrug resistance (MDR), high transmissibility in healthcare settings, and significant mortality. The World Health Organization classifies it as a ‘critical priority’ fungal pathogen. Conventional infection control paradigms, often applying a ‘one-size-fits-all’ approach, have struggled to contain *C. auris* due to its unique environmental resilience and transmission dynamics. This review departs from traditional linear analyses and proposes a novel, scenario-based framework to deconstruct the complex challenges of *C. auris* management. We dissect the distinct transmission dynamics and control vulnerabilities across four high-risk clinical scenarios: the intensive care unit (ICU), long-term care facility (LTCF), high-risk surgical ward, and outpatient settings. Building on this analysis, we introduce the ‘*C. auris* Integrated Control Bundle,’ a multi-layered, adaptable toolkit combining patient-level, facility-level, and system-level interventions. This framework provides clinicians and infection preventionists with a practical, evidence-based paradigm to design and implement setting-specific, resource-optimized strategies against this resilient pathogen. We also review current diagnostic and therapeutic challenges, emphasizing the urgent need for rapid diagnostics and novel treatment options. By addressing key unanswered questions, this review aims to guide future research and strengthen the global response to *C. auris*.

## 1 Introduction: the emergence of a formidable foe

The emergence of *Candidozyma auris* as a WHO-designated “critical priority” pathogen has exposed the limitations of conventional, one-size-fits-all infection control paradigms. Although first formally described in 2009, retrospective analyses have identified isolates dating back to at least 1996 (Du et al., 2020). For over a decade, its true prevalence was masked by diagnostic misidentification, as conventional phenotypic methods often mislabeled *C. auris* as closely related species like *Candida haemulonii* or even other common yeasts (Kathuria et al., 2015; Kordalewska and Perlin, 2019). This prolonged period of cryptic circulation likely facilitated its silent establishment in healthcare environments worldwide, allowing it to become a globally entrenched pathogen before its unique threat was fully recognized.

Its rapid global spread is driven by a unique combination of multidrug resistance, prolonged skin colonization, and remarkable environmental persistence. The core challenge lies not in a lack of interventions, but in the failure of generic guidelines to account for the highly variable interplay of these factors across different clinical environments. A deeper understanding requires deconstructing the problem to its fundamental drivers: the core mechanisms of pathogen colonization, environmental persistence, and inter-patient transmission.

Recognizing that the expression and interplay of these core mechanisms are highly context-dependent, a scenario-based analysis emerges as a necessary methodological approach. The present review operationalizes this approach by systematically analyzing the distinct transmission dynamics and control vulnerabilities across four high-risk clinical scenarios: the intensive care unit, the long-term care facility, the high-risk surgical ward, and the outpatient hemodialysis unit. For each scenario, we delineate the specific transmission pathways and identify key control points. Furthermore, we synthesize these findings into an ‘Integrated Control Bundle,’ a structured, multi-level framework designed to be adapted to the specific risk profile of each clinical environment. The overarching aim is to move beyond descriptive summaries and provide a context-specific paradigm for the containment of *C. auris* colonization and transmission.

## 2 The pathogen: understanding *Candidozyma auris*

*C. auris* possesses a unique combination of biological attributes that underpin its success as a nosocomial pathogen. Its resilience, genetic diversity, and sophisticated virulence mechanisms enable it to thrive in healthcare environments and resist clinical interventions.

### 2.1 Microbiological characteristics and phylogenetics

Genomically, *C. auris* is a haploid yeast characterized by significant genetic diversity and a highly plastic karyotype, with chromosome numbers varying from five to seven among isolates (Bravo Ruiz et al., 2019; Narayanan et al., 2021). This karyotypic variability allows for rapid adaptation to environmental stressors, which may contribute to its pathogenic versatility (Bravo Ruiz et al., 2019; Narayanan et al., 2021). The species is phylogenetically divided into six distinct clades (Clade I-VI), each with a primary geographic origin and characteristic antifungal susceptibility profile (Chowdhary et al., 2023; da Silva et al., 2025). For example, Clade I is predominant in South Asia, Clade II in East Asia, Clade III in Africa, Clade IV in South America, and the most recently discovered Clade V in Iran (Chowdhary et al., 2023; da Silva et al., 2025), and the most recently described Clade VI in Singapore (Suphavilai et al., 2024). Whole-genome sequencing (WGS) has become an indispensable tool for tracking the global and local transmission of these clades, confirming the clonal spread of strains across different regions and within healthcare facilities (Burrack et al., 2022; Mitchell et al., 2024).

Furthermore, *C. auris* is highly thermotolerant, capable of growing at temperatures up to 42°C, a trait confirmed to be a key virulence factor for survival at human body temperature and potentially linked to its emergence in a warming climate (Cha et al., 2025; Cosio et al., 2025). This thermotolerance distinguishes *C. auris* from many other *Candida* species and may have facilitated its adaptation to mammalian hosts.

### 2.2 Clade-specific cell aggregation

A notable phenotypic characteristic that varies among *C. auris* clades is the tendency to form cell aggregates. Isolates from Clade I (South Asia), Clade III (Africa), and the recently identified Clade V (Iran) are often “aggregating,” meaning the daughter cells fail to separate completely after budding, resulting in large clumps of cells that are difficult to disperse (Chatterjee et al., 2015; Chow et al., 2020). In contrast, isolates from Clade II (East Asia) and Clade IV (South America) are typically “non-aggregating,” growing as individual or paired yeast cells (Chatterjee et al., 2015). Initial reports on the newly described Clade VI suggest it shares this non-aggregating phenotype (Suphavilai et al., 2024).

This difference is largely attributed to defects in cell wall remodeling, specifically related to the activity of chitinase enzymes. In aggregating strains, mutations or altered expression of genes encoding key chitinases (such as *CHS2*) impair the degradation of the chitin-rich primary septum that separates mother and daughter cells. This failure of cell separation leads to the characteristic clumping phenotype (Muñoz et al., 2018; Rossato and Colombo, 2018).

While seemingly a cellular defect, this aggregation has significant clinical and biological implications. For clinical laboratories, the aggregates complicate accurate colony-forming unit (CFU) counting and can interfere with standard antifungal susceptibility testing (AST), as the dense cell clusters may impede drug penetration and lead to falsely elevated resistance results (Bidaud et al., 2018). From a pathogenic standpoint, aggregation may confer a survival advantage. These cell clumps can be considered a rudimentary form of biofilm, potentially offering protection against phagocytosis by host immune cells and enhancing adherence to surfaces like medical catheters (Rossato and Colombo, 2018; Huang et al., 2021). This may contribute to the observation that aggregating clades, particularly Clade I, are frequently associated with large-scale healthcare-associated outbreaks worldwide.

### 2.3 Limited filamentation capacity

The capacity of *C. auris* for filamentation is a subject of debate and appears significantly more restricted compared to other pathogenic *Candida* species. Unlike *C. albicans*, which readily forms true hyphae as a key virulence trait, *C. auris* does not form true hyphae (Borman et al., 2016). However, some strains can exhibit a limited switch to pseudohyphal or elongated yeast forms, particularly under specific environmental stresses such as nutrient limitation or exposure to certain antifungal agents (Bravo Ruiz et al., 2020; Fan et al., 2021). This limited morphological plasticity may still play a role in virulence, potentially contributing to tissue invasion and the structural integrity of biofilms. Nevertheless, its overall contribution to pathogenesis is considered less significant than the robust yeast-to-hypha transition seen in *C. albicans*, and it is not a universal feature across all *C. auris* isolates (Jackson et al., 2019).

### 2.4 Biological drivers of colonization and transmission

The success of *C. auris* as a nosocomial pathogen stems from a combination of key biological traits. Its multidrug resistance, robust biofilm formation, and remarkable environmental resilience are not isolated features; they work together as an integrated system that drives patient colonization, environmental persistence, and inter-patient transmission. Understanding this interplay is essential to explain why traditional control measures often fail and why a scenario-based framework is necessary.

### 2.5 Antifungal resistance: creating the opportunity for colonization

Antifungal resistance in *C. auris* provides a critical advantage for establishing its presence in a host. The near-universal resistance to fluconazole, a widely used antifungal, is particularly significant (Kordalewska and Perlin, 2019). In clinical settings, patients receiving fluconazole are cleared of susceptible *Candida* species, which inadvertently creates an open ecological niche. *C. auris*, unaffected by the drug, can then colonize these patients with little competition, turning them into sources for further spread (Schelenz et al., 2016).

This resistance is primarily driven by mutations in the *ERG11* gene, which encodes the drug’s target, and by the overexpression of efflux pumps (e.g., *CDR1*, *MDR1*) that expel the drug from the cell (Chowdhary et al., 2018; Kean et al., 2020). The clinical picture is further complicated by emerging resistance to echinocandins, the first-line therapy for invasive candidiasis. This resistance, often developing during treatment through mutations in the *FKS1* gene (e.g., S639F), can lead to treatment failure and prolonged patient colonization, thereby widening the window for hospital-acquired transmission (Eyre et al., 2018; Biagi et al., 2019). The emergence of pan-resistant isolates, which are non-susceptible to all three major antifungal classes, represents a worst-case scenario, making both treatment and decolonization extremely difficult (Ostrowsky et al., 2020). Importantly, while many mutations in genes such as ERG11 have been reported as potentially related to drug resistance in *C. auris*, most of these initial findings were from small-sample studies and thus lacked rigorous statistical support. A recent genome-wide association study (GWAS) analyzing 387 global *C. auris* isolates provided the first population-level statistical validation of resistance-associated genomic variants (Wang and Xu, 2024). This study revealed that the genetic basis of resistance is far more diverse and complex than previously understood. The GWAS confirmed that the majority of statistically significant resistance-associated SNPs are clade-specific and located in genes or intergenic regions not previously linked to antifungal resistance. In fact, only four SNPs in ERG11 and FKS1 were found to be shared between clades, with the FKS1 S639F/P substitution being the most prominent example with prior experimental confirmation. These findings indicate that *C. auris* has independently evolved diverse resistance mechanisms within each clade, which complicates the development of universal molecular diagnostics and underscores the critical need for continuous genomic surveillance.

### 2.6 Biofilm formation: the anchor for persistence and transmission

If drug resistance creates the opportunity for colonization, biofilm formation is the mechanism that allows *C. auris* to anchor itself for long-term survival and spread. Biofilms are structured communities of cells encased in a protective matrix, serving two main purposes in the transmission cycle.

First, the biofilm matrix acts as a physical shield, limiting the penetration of antifungal drugs and protecting the yeast from the host immune system. This can increase drug tolerance dramatically, making biofilm-related infections, such as those on catheters, highly resistant to standard therapy (Li et al., 2023).

Second, and more importantly for transmission, biofilms allow *C. auris* to adhere firmly to both patient skin and inanimate surfaces. On patients, this leads to persistent skin colonization, creating a continuous source for shedding the pathogen into the environment (Horton et al., 2020; Sexton et al., 2021). On hospital equipment, biofilms form resilient communities on plastics (e.g., catheters) and dry surfaces (e.g., bed rails), turning them into persistent environmental reservoirs. These contaminated surfaces then become critical points for transmission via the hands of healthcare workers or direct patient contact (Welsh et al., 2017). The robustness of these biofilms explains why eradicating *C. auris* from the healthcare environment is so challenging and why the complete physical removal of the colonized device (e.g., central venous catheters, urinary catheters) is often necessary to resolve the infection (Kean et al., 2020).

## 3 Clinical scenarios: transmission and management challenges

**Figure 1:**
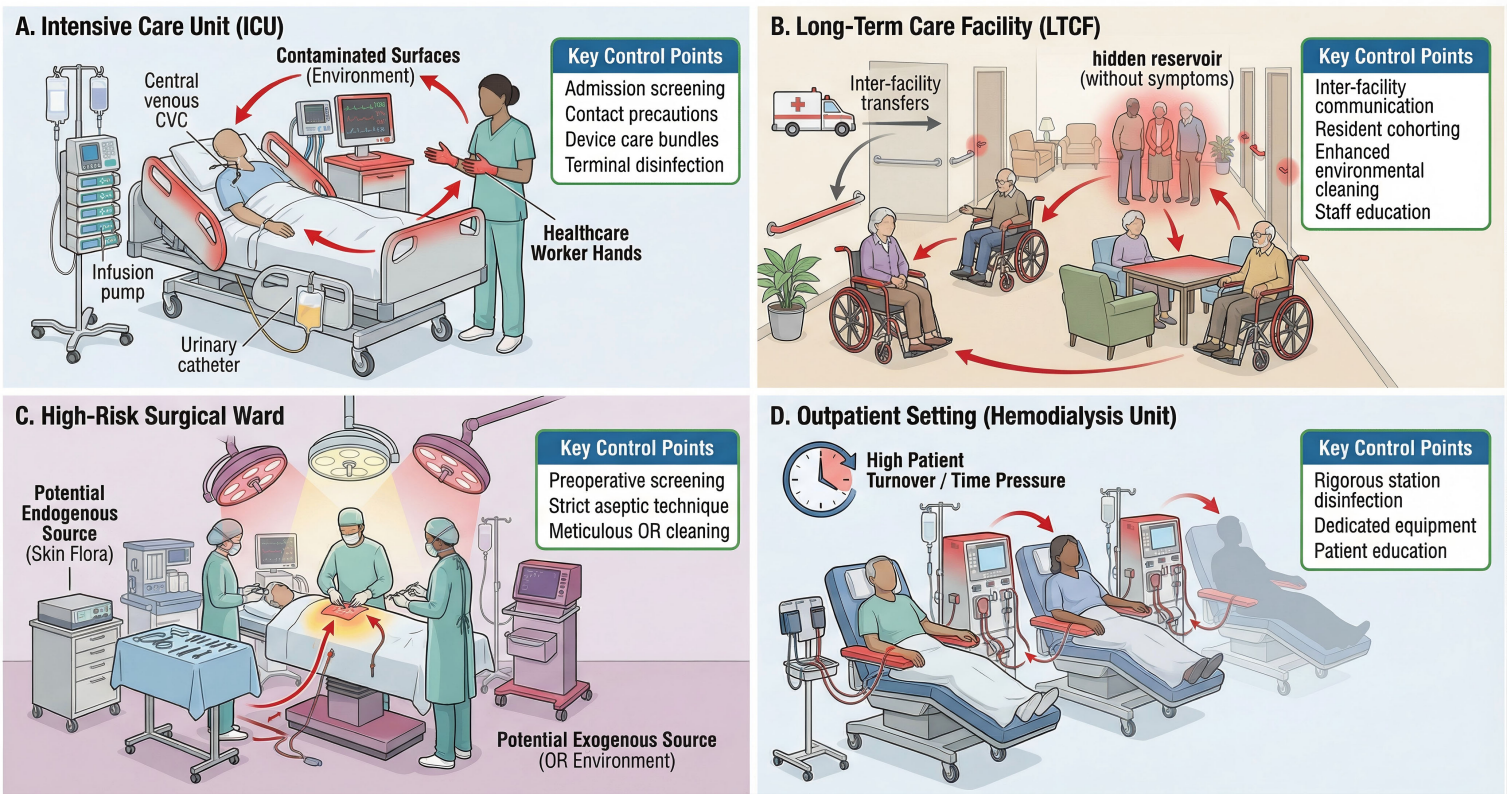
Transmission dynamics and key control points for *C. auris* across four clinical scenarios. This diagram provides a comparative overview of *C. auris* transmission pathways and highlights scenario-specific control points in four distinct healthcare settings. **(A)** Intensive Care Unit (ICU): Transmission is rapid, fueled by frequent healthcare worker-patient interactions, high density of invasive devices (e.g., CVCs, ventilators), and extensive environmental contamination of high-touch surfaces. Key control points include active surveillance screening on admission, strict contact precautions, device care bundles, and terminal disinfection with sporicidal agents. **(B)** Long-Term Care Facility (LTCF): Transmission is often silent and persistent, driven by communal living, shared equipment, and frequent resident transfers, which create a regional reservoir. Control hinges on inter-facility communication, resident cohorting, and enhanced, routine environmental cleaning. **(C)** High-Risk Surgical Ward: Transmission can occur via the patient’s endogenous flora or a contaminated operating room environment, leading to severe postoperative infections. Control focuses on preoperative screening for high-risk patients, strict aseptic technique, and meticulous cleaning of the surgical environment. **(D)** Outpatient Setting (e.g., Hemodialysis Unit): Transmission is facilitated by repeated contact with shared equipment (e.g., dialysis machines, chairs) and high patient turnover. Key control points include rigorous disinfection of stations between patients, designation of dedicated equipment for colonized individuals, and patient education.

To effectively combat *C. auris*, control strategies must be tailored to the specific environments where it proliferates. The transmission dynamics, patient vulnerabilities, and management challenges differ substantially across various healthcare settings. This section analyzes the unique epidemiology and control considerations in four key clinical scenarios (**Figure 1**).

### 3.1 Scenario 1: the critically ill patient in the intensive care unit

Scene description: The ICU represents a high-risk epicenter for *C. auris* transmission. Patients are critically ill, often with multiple comorbidities, suppressed immune systems, and require numerous invasive medical devices for life support (Rathod et al., 2025).

Transmission characteristics: Transmission in the ICU is rapid and efficient. The high density of vulnerable patients, frequent healthcare worker interactions, and ubiquitous use of invasive devices create conditions favorable for outbreaks. Indwelling devices such as central venous catheters (CVCs), urinary catheters, mechanical ventilators, and even mechanical circulatory support devices serve as primary conduits for infection (Fan et al., 2025). The environment itself becomes a persistent reservoir, with *C. auris* contaminating bed rails, infusion pumps, and other shared equipment. Failure in hand hygiene allows healthcare workers’ hands to become transiently contaminated, facilitating the cross-transmission of pathogens between the patient zone and the inanimate environment (Stiefel et al., 2011; Doran et al., 2025). Environmental contamination is often extensive; for instance, in one high-risk setting, *C. auris* was recovered from all sampled handrails associated with colonized patients, underscoring the role of frequently touched surfaces as persistent reservoirs (Sexton et al., 2021).

Management Challenges: A primary challenge in the ICU is distinguishing between benign colonization and life-threatening invasive infection. Critically ill patients often have multiple reasons for clinical deterioration, making the diagnosis of candidemia difficult and often delayed. This is particularly perilous as the progression from colonization to invasive infection can be rapid, with one study finding that approximately one in eight colonized patients develop an invasive infection, facing a significantly higher mortality rate (65.9% vs. 33.1%) [26]. The high prevalence of MDR strains in this setting further complicates empirical treatment, rendering many standard antifungal therapies ineffective [7]. Clinicians face the dilemma of when to initiate empirical antifungal therapy in colonized patients who develop fever or sepsis, as delayed treatment can be fatal, yet unnecessary treatment contributes to further resistance development and drug toxicity.

Control Points: Control in the ICU hinges on a multi-pronged approach. This includes strict adherence to contact precautions and hand hygiene. Active surveillance screening on admission is crucial for early identification of colonized patients, allowing for prompt implementation of isolation or cohorting measure (Medioli et al., 2025**;**Myrou et al., 2025). Bundled care for invasive devices (e.g., catheter care bundles) is essential to prevent entry-point infections (El-Sokkary et al., 2025). Finally, terminal cleaning and disinfection of patient rooms and equipment with a sporicidal agent are necessary to break the cycle of environmental contamination (Li et al., 2025; Tarka et al., 2025). Daily environmental cleaning with appropriate disinfectants, combined with regular audits of cleaning practices, has been shown to significantly reduce environmental burden and subsequent transmission rates.

### 3.2 Scenario 2: the resident in a long-term care facility

Scene description: LTCFs, including skilled nursing facilities, are increasingly recognized as major reservoirs for *C. auris* and hubs for regional spread. Residents are often elderly, have multiple chronic conditions, and may be colonized for extended periods, often asymptomatically (Ahmad and Alfouzan, 2021; Sanyaolu et al., 2022).

Transmission characteristics: Transmission in LTCFs is often silent and persistent (Zhang et al., 2025a). The high-touch, communal living environment and frequent resident transfers between facilities facilitate widespread dissemination (Sexton et al., 2021). Colonized residents act as a “hidden reservoir,” continuously shedding the organism into their environment (Sexton et al., 2021). Staff, who may care for multiple residents, can inadvertently spread *C. auris* if hand hygiene is suboptimal. Shared rehabilitation equipment and common areas also contribute to cross-contamination (Sexton et al., 2021). The transfer of colonized residents from LTCFs to acute care hospitals is a primary driver of hospital outbreaks. In some regions, LTCFs have become endemic for *C. auris*, with colonization rates exceeding 10% of residents, creating a persistent source for regional spread (Barbian et al., 2025).

Management challenges: The challenges in LTCFs are distinct from those in acute care. The goal is often containment and prevention of transmission rather than cure of colonization. Large-scale screening is resource-intensive, and effective decolonization strategies for chronically colonized residents remain elusive; while chlorhexidine (CHG) bathing is often used, its efficacy is limited and raises concerns about the potential for reduced susceptibility (Frías-De-León et al., 2025). Maintaining high levels of staff adherence to infection control practices over the long term can be difficult, particularly in facilities with high staff turnover and limited resources. However, effective communication regarding patient colonization status during inter-institutional transfers remains a major logistical barrier in regional control strategies (Ray et al., 2025). The social and psychological impact of prolonged isolation on elderly residents must also be balanced against infection control needs.

Control points: Key strategies for LTCFs include active surveillance to identify colonized residents and inter-facility communication protocols upon resident transfer. Cohort nursing, where specific staff are assigned to care only for colonized residents, can limit spread. Enhanced environmental cleaning, particularly of high-touch surfaces and shared equipment, is critical. Educating staff, residents, and families about the importance of hand hygiene and other precautions is also a cornerstone of control (Mody et al., 2025). Some facilities have successfully implemented real-time electronic health record (EHR) alerts, often referred to as “colonization flags,” which automatically notify clinicians of a patient’s multidrug-resistant organism (MDRO) colonization status upon admission or transfer. This mechanism is crucial for improving the continuity of infection control measures and has been shown to enhance timely clinical interventions and reduce adverse outcomes (Dzintars et al., 2021; Ray et al., 2025).

### 3.3 Scenario 3: the high-risk surgical patient

Scene description: Patients undergoing major surgery, such as cardiothoracic or abdominal procedures, represent another vulnerable population. They experience significant physiological stress, breaches in skin integrity, and often require multiple invasive devices during their perioperative course.

Transmission characteristics: While less common than in ICUs, the physiological stress and invasive procedures inherent to major surgeries, such as cardiothoracic and abdominal procedures, place these patients at high risk for healthcare-associated *C. auris* acquisition and subsequent invasive infection, including SSIs and candidemia (Alowais et al., 2025; Rathod et al., 2025). Transmission can occur from the patient’s own skin flora if they are pre-colonized, or from a contaminated operating room environment or contaminated hands of the surgical team (Curless et al., 2025). Post-operative care in a surgical ICU carries similar risks to the general ICU scenario. Surgical patients who are colonized with *C. auris* prior to their procedure face a significantly elevated risk of developing invasive infection, particularly if they undergo abdominal surgery or require prolonged postoperative mechanical ventilation (Alowais et al., 2025).

Management challenges: A primary challenge is the high mortality associated with postoperative *C. auris* SSIs and bloodstream infections (Kim et al., 2024; Wang et al., 2024). Diagnosing these infections can be difficult, as postoperative fever is common and non-specific. One study found that recent abdominal surgery was an independent predictor for progression from colonization to invasive infection, highlighting the vulnerability of this patient population (Alowais et al., 2025). The presence of surgical drains, which can serve as conduits for infection, further complicates management. Additionally, current guidelines lack robust evidence to support routine antifungal prophylaxis specifically for *C. auris*-colonized surgical patients, a gap that complicates management and raises concerns about accelerating antifungal resistance (Kim et al., 2024; Wang et al., 2024).

Control points: Prevention is paramount. For high-risk surgeries, preoperative *C. auris* colonization screening should be considered, especially for patients transferred from high-prevalence settings like LTCFs. Strict adherence to aseptic technique during surgery and wound care is fundamental. Meticulous environmental cleaning of operating rooms between cases is also critical (Ahmad and Alfouzan, 2021; Curless et al., 2025). For patients known to be colonized, implementing enhanced contact precautions throughout the perioperative period is a key measure. Some institutions have adopted protocols for preoperative CHG bathing for colonized patients, though the evidence for its efficacy in preventing postoperative *C. auris* infections is still emerging (Frías-De-León et al., 2025).

### 3.4 Scenario 4: outpatient settings (e.g., dialysis centers, wound care clinics)

Scene description: Outpatient settings that provide chronic care to medically complex patients, such as hemodialysis centers, oncology clinics, and wound care centers, also pose a risk for *C. auris* transmission. These patients often have compromised immune systems or require long-term vascular access (Hassoun-Kheir et al., 2025).

Transmission characteristics: Frequent and repeated patient contact with shared medical environments and equipment facilitates transmission. In hemodialysis centers, dialysis machines and chair areas can become contaminated, posing a risk to subsequent patients. Patients may be chronically colonized, serving as a persistent source of transmission during their frequent visits (Montoya Urrego et al., 2022; Kurutz et al., 2025). The intensive scheduling inherent to hemodialysis units creates time constraints that pose a significant barrier to achieving optimal terminal disinfection between patient sessions (Ngema et al., 2025).

Management challenges: Outpatient facilities often have different environmental cleaning standards and infection control resources compared to hospitals (Bringhurst, 2019; Penna et al., 2025). Ensuring consistent adherence to protocols among a mobile patient population and rotating staff can be difficult. The high mobility of patients makes contact tracing and outbreak investigation complex (Knighton et al., 2025). Additionally, patients may receive care at multiple outpatient facilities, creating opportunities for cross-facility transmission that are difficult to track and control.

Control points: Control strategies must be adapted to the outpatient workflow. This includes designating dedicated chairs or treatment stations for colonized patients when feasible. Rigorous cleaning and disinfection of patient care stations and equipment between each patient is non-negotiable (Trocino et al., 2025). Patient education about hand hygiene and self-care is critical (Knighton et al., 2025). Finally, establishing robust communication mechanisms between outpatient clinics and other healthcare facilities where patients may receive care (e.g., hospitals) is essential for ensuring continuity of infection control measures. Visual cues and color-coded systems have been successfully implemented in various high-risk clinical settings, such as critical care and procedural units, demonstrating efficacy in reducing contamination and improving adherence to complex protocols. This principle is transferable to high-risk outpatient environments like hemodialysis units (George et al., 2021).

## 4 Diagnosis and treatment: navigating clinical challenges

Accurate and timely diagnosis of *C. auris* colonization and infection is critical for guiding appropriate treatment and implementing infection control measures. However, significant challenges exist in both identification and therapeutic management.

### 4.1 Diagnostic approaches

Traditional culture-based methods can misidentify *C. auris*, as it may be confused with other *Candida* species using conventional biochemical tests. Matrix-assisted laser desorption/ionization time-of-flight mass spectrometry (MALDI-TOF MS) has emerged as a reliable method for species-level identification, provided that reference databases include *C. auris* spectra (Hsu and Yassin, 2025). Molecular methods, including PCR-based assays and whole-genome sequencing, offer high specificity and can provide additional information about clade type and resistance mechanisms (Korsten et al., 2025).

For surveillance screening, axillary and inguinal swabs are the preferred sample sites (Myrou et al., 2025; Tan et al., 2025), as these areas typically harbor the highest fungal burden. Rapid molecular diagnostic tests suitable for near-patient use are urgently needed to enable real-time decision-making in clinical settings (Banik et al., 2024). The necessity of admission screening is underscored by studies showing a large hidden reservoir of multidrug-resistant organism (MDRO) carriers, particularly among patients transferred from post-acute care settings, many of whom have no known prior colonization history (Schechner et al., 2025).

### 4.2 Therapeutic challenges

Treatment of invasive *C. auris* infections is complicated by high rates of antifungal resistance. Echinocandins (e.g., caspofungin, micafungin, anidulafungin) are generally considered first-line therapy for invasive infections, but resistance can emerge during treatment (Cornely et al., 2025). For azole-resistant strains, which constitute the majority of isolates, fluconazole and other azoles are ineffective. Amphotericin B formulations may be used for echinocandin-resistant isolates, though resistance to this agent has also been reported (Salmanton-García et al., 2026).

The emergence of pan-drug resistant strains leaves clinicians with few or no effective treatment options (Rhodes et al., 2024). In such cases, combination antifungal therapy or the use of investigational agents may be considered, though evidence to support these approaches is limited (Wang et al., 2024).

Prompt implementation of source control measures, such as the definitive removal of infected foreign bodies (e.g., central venous catheters) and timely drainage of localized collections (e.g., abscesses), is paramount for clinical success in invasive infections. Early consultation with infectious disease specialists and clinical microbiologists is essential for optimizing treatment strategies and interpreting susceptibility testing results (O’Grady et al., 2023; Bourassa-Blanchette et al., 2024).

### 4.3 The pipeline of new antifungals

In response to the escalating threat of multidrug resistance, the antifungal development pipeline has advanced considerably, offering promising new options against *C. auris*. Several novel agents with different mechanisms of action are in late-stage clinical development or have recently been approved.

Rezafungin, a next-generation echinocandin administered once-weekly, has been approved for treating candidemia and invasive candidiasis. In the pivotal ReSTORE Phase 3 trial, rezafungin demonstrated non-inferiority to daily caspofungin, with a Day 14 global cure rate of 56.5% versus 57.3% for caspofungin, and a comparable Day 30 all-cause mortality rate of 25.2% (Thompson et al., 2023). Its long half-life, allowing for a 400 mg loading dose followed by 200 mg once-weekly, offers a significant advantage in clinical practice by simplifying treatment regimens, although no *C. auris* patients were included in this specific trial.

Ibrexafungerp, the first in a new class of triterpenoid antifungals, functions as a glucan synthase inhibitor but is uniquely available in an oral formulation. It has shown potent *in vitro* activity and clinical efficacy against *C. auris*, providing a much-needed oral treatment option for step-down therapy. In the open-label CARES study, which enrolled 18 patients with *C. auris* candidemia or invasive candidiasis, oral ibrexafungerp (750 mg BID for 2 days, then 750 mg QD) was used as primary or step-down therapy (Wiederhold et al., 2021). Given the lack of oral options for echinocandins, ibrexafungerp fills a critical gap in the transition from intravenous to oral therapy for stable patients (Ghannoum et al., 2020).

Perhaps the most anticipated is Fosmanogepix, a first-in-class agent that targets the fungal enzyme Gwt1. This novel mechanism of action makes it highly effective against many multidrug-resistant fungi, including *C. auris*. A Phase 2 proof-of-concept study demonstrated a high treatment success rate of 80% (16/20 patients) at the end of therapy, with a Day 30 survival rate of 85%. Notably, this trial included 9 patients specifically with *C. auris* infections (Pappas et al., 2023), positioning fosmanogepix as a critical future tool against pan-resistant strains.

Beyond these leading candidates, preclinical studies are exploring other innovative strategies. Silver nanoparticles (AgNPs) have demonstrated potent *in vitro* activity against *C. auris*, with minimum inhibitory concentrations (MICs) as low as 0.5–4 μg/mL against planktonic cells (Vazquez-Munoz et al., 2020). Critically, AgNPs also show strong antibiofilm properties; when functionalized onto silicone elastomer surfaces—a material commonly used in medical devices—they inhibited *C. auris* biofilm formation by over 80%. Among natural products, essential oils have emerged as promising candidates. In a screening of 21 essential oils, those derived from lemongrass (Cymbopogon citratus), clove bud (Syzygium aromaticum), and cinnamon bark (Cinnamomum zeylanicum) exhibited the strongest fungicidal activity against *C. auris* at concentrations considered safe for topical use (Parker et al., 2022). Notably, C. zeylanicum bark oil demonstrated synergistic interactions when combined with conventional antifungal drugs against 10 clinical *C. auris* strains (Di Vito et al., 2023). While these approaches are still far from systemic clinical use, they hold particular promise for topical decolonization and surface disinfection strategies—areas directly relevant to infection prevention. The exploration of combination therapy using existing drugs is also a key strategy. The combination of micafungin with amphotericin B has shown *in vitro* synergy against pan-resistant *C. auris* isolates, suggesting a potential second-line regimen when monotherapy fails (O’Brien et al., 2020). More innovatively, drug repurposing studies have demonstrated that fluvastatin, a commonly used cholesterol-lowering statin, exhibits synergistic effects with azole antifungals (posaconazole, voriconazole, isavuconazole) in 70–90% of 21 clinical *C. auris* isolates, with posaconazole/fluvastatin achieving synergy in 19/21 (90%) isolates and a significant 8-fold reduction in posaconazole MIC (Halliday et al., 2023). These findings open entirely new avenues for repurposing existing, well-characterized drugs against this formidable pathogen.

## 5 The *C. auris* integrated control bundle

**Figure 2:**
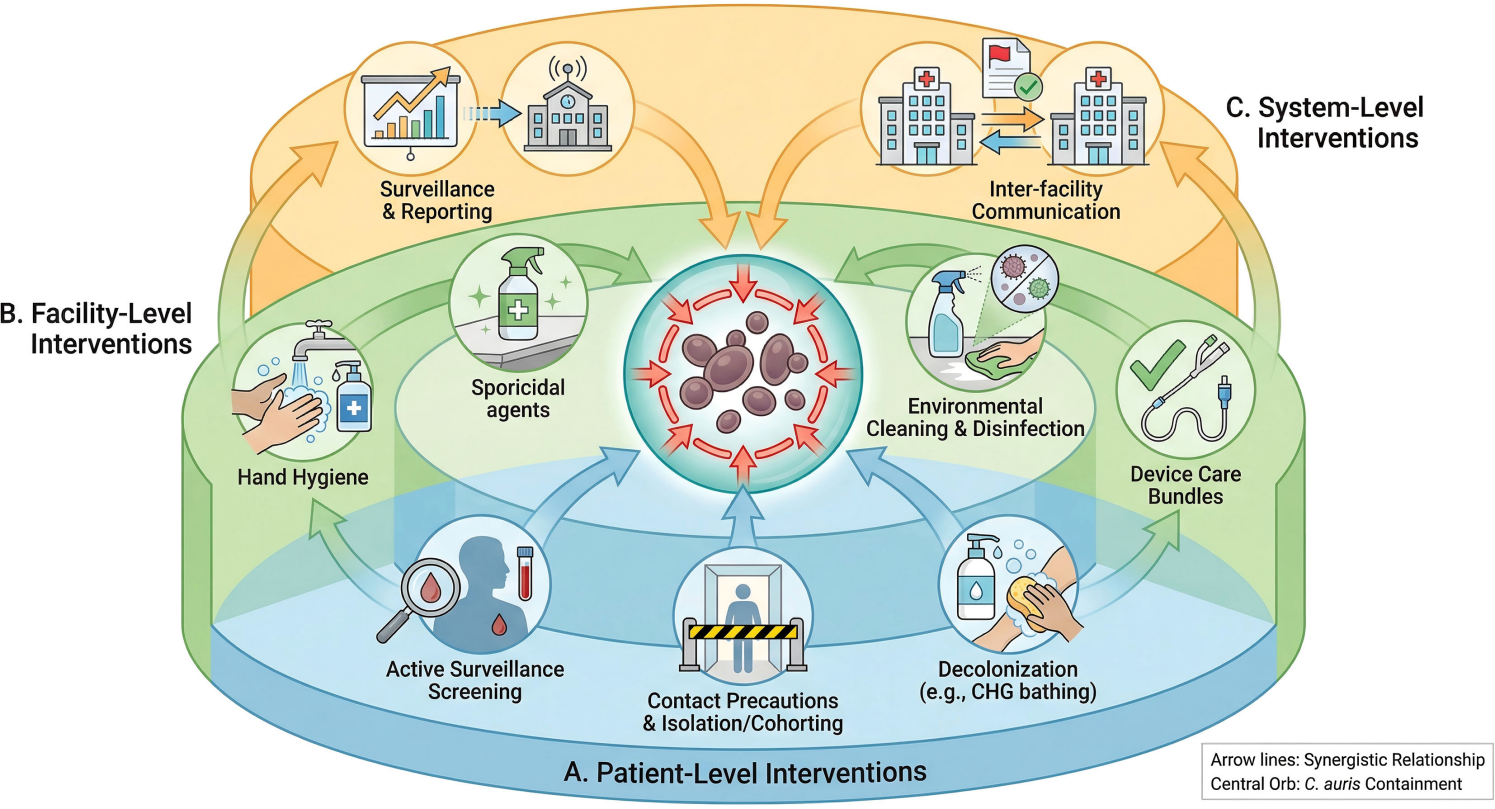
The *C. auris* integrated control bundle. This figure presents a multi-tiered, adaptable framework for the prevention and control of *C. auris*. The bundle is organized into three synergistic levels. **(A)** Patient-Level Interventions: These are the foundational measures focused on the individual, including risk-based active surveillance screening to identify carriers, prompt implementation of contact precautions and isolation/cohorting, and consideration of decolonization strategies (e.g., CHG bathing) in specific high-risk or outbreak situations. **(B)** Facility-Level Interventions: This layer forms the backbone of environmental control, encompassing meticulous hand hygiene, enhanced environmental cleaning and disinfection with effective agents (e.g., sporicidals), and strict adherence to device care bundles to prevent healthcare-associated infections. **(C)** System-Level Interventions: This overarching layer ensures a coordinated response across the healthcare system. It includes robust surveillance and mandatory reporting to public health authorities and, critically, clear and consistent inter-facility communication protocols to flag a patient’s colonization status during transfers, thereby preventing silent introductions into new facilities. .

A static, universal protocol is insufficient to control *C. auris*. We propose an Integrated Control Bundle, a multi-tiered framework that combines core infection prevention principles with scenario-specific actions. The bundle is organized into three levels: patient-level, facility-level, and system-level interventions. The novelty of this approach lies in its adaptability, allowing healthcare institutions to construct a tailored defense based on their specific patient populations and environments (**Figure 2**).

### 5.1 Patient-level interventions

Patient-level interventions focus on identifying carriers and reducing the risk of transmission and infection at the individual level.

- Screening: Risk-based active surveillance screening is the cornerstone of early detection. Screening should be prioritized for patients with known risk factors, such as admission from high-prevalence facilities (especially LTCFs), prior hospitalization in outbreak regions, or close contact with known cases (Myrou et al., 2025). Axillary and inguinal sites are the preferred swab collection sites (Myrou et al., 2025). The availability of rapid molecular diagnostic methods (e.g., PCR-based assays) is critical for timely results that can inform patient placement and cohorting decisions (Harbarth et al., 2006; Qian et al., 2025).
- Isolation and cohorting: Once identified, colonized or infected patients should be placed under contact precautions in single rooms. When single rooms are unavailable, cohorting patients with dedicated staff and equipment is the next-best strategy. This is particularly critical in ICU and LTCF outbreak settings to prevent further spread (Li et al., 2025; Sussenbach et al., 2025).
- Decolonization: The role of decolonization remains a contentious topic (Frías-De-León et al., 2025). Daily chlorhexidine (CHG) bathing is a common practice aimed at reducing the *C. auris* burden on the skin. However, its efficacy in complete eradication is limited, and prolonged use raises concerns about the potential for reduced CHG susceptibility or resistance (Johnson et al., 2021; Frías-De-León et al., 2025). Current evidence does not support routine decolonization for all colonized patients, but it may be considered as part of a broader, more intensive control strategy during outbreaks, after careful risk-benefit assessment (Gussin et al., 2025; Salmanton-García et al., 2026).

### 5.2 Facility-level interventions

Facility-level interventions form the backbone of environmental control and prevention of cross-transmission.

- Hand hygiene: Meticulous hand hygiene is the single most important measure to prevent transmission. Alcohol-based hand sanitizers are effective against *C. auris* and should be readily available. Auditing and providing feedback on healthcare worker hand hygiene compliance is essential for maintaining high adherence rates (Rathod et al., 2025).
- Environmental cleaning and disinfection: Given its tenacious survival in the environment, routine cleaning is inadequate. Daily and terminal cleaning of patient rooms and shared equipment must be performed using disinfectants proven to be effective against *C. auris*. Environmental disinfection protocols for high-consequence pathogens require agents effective against C. difficile spores and validated efficacy against emerging threats like *C. auris* (Boyce, 2024*;*Frías-De-León et al., 2025*)*. Novel technologies such as ultraviolet (UV-C) light and vaporized hydrogen peroxide (VHP) can be valuable adjuncts for terminal room disinfection, especially in outbreak situations, though their implementation may be limited by cost and operational complexity (Frías-De-León et al., 2025).
- Device care: Strict adherence to aseptic technique during insertion and maintenance of invasive medical devices is paramount. Care bundles for central venous catheters, urinary catheters, and ventilators have been proven to reduce the risk of device-associated infections and should be rigorously enforced (Buetti et al., 2022).

### 5.3 System-level interventions

System-level interventions are necessary to ensure a coordinated response across the healthcare continuum.

- Surveillance and reporting: Establishing robust local and regional surveillance networks is critical for monitoring the emergence and spread of *C. auris*. Mandatory reporting of cases to public health authorities facilitates better understanding of regional epidemiology and enables coordinated responses (Tan et al., 2025; Salmanton-García et al., 2026).
- Inter-facility communication: Clear and consistent communication during patient transfers is essential for preventing the silent introduction of *C. auris* into new facilities. A patient’s colonization or infection status must be prominently flagged in their medical record and communicated to the receiving facility prior to transfer. This is especially important for transfers between LTCFs and acute care hospitals (Resong et al., 2025).

### 5.4 A scenario-based control bundle proposal

The strength of the Integrated Control Bundle lies in its application to specific clinical scenarios. The following table provides a framework for prioritizing and combining interventions based on the risk profile of each setting (**Table 1**).

**Table 1:** A scenario-based decision-making table for C. auris infection prevention and control.

Intervention level	ICU scenario	LTCF scenario	High-risk surgical scenario	Outpatient (dialysis) scenario
Patient-Level	Core: Admission screening, Contact precautions, Daily CHG bathing.	Core: Admission screening, Contact precautions. Supplemental: Periodic screening, Cohorting.	Core: Preoperative screening for high-risk transfers. Supplemental: Preoperative CHG bathing.	Core: Routine screening for all patients. Supplemental: Patient hygiene education.
Facility-Level	Core: Strict hand hygiene, Sporicidal daily & terminal cleaning, Dedicated equipment. Supplemental: UV-C/VHP for terminal cleaning.	Core: Strict hand hygiene, Enhanced environmental cleaning of common areas. Supplemental: Dedicated staff for cohorts.	Core: Meticulous OR cleaning, Strict aseptic technique, Instrument sterilization.	Core: Stringent station disinfection between patients, Dedicated equipment.
System-Level	Core: Immediate reporting to infection control & public health.	Core: Mandatory flagging & communication on transfer.	Core: Communication with transferring facility about patient status.	Core: Communication with hospitals regarding patient status.

This scenario-based table serves as a decision-making tool, allowing infection prevention teams to escalate or de-escalate measures based on the local epidemiology and specific setting, thereby optimizing resource allocation and maximizing the effectiveness of control efforts.

## 6 Unanswered questions and future directions

Despite rapid advances in our understanding of *C. auris*, significant knowledge gaps remain. Addressing these questions is critical for developing the next generation of tools and strategies to control this pathogen.

How can we effectively decolonize patients? One of the most pressing challenges is the lack of a proven, safe, and effective regimen for eradicating *C. auris* colonization. While CHG is widely used, its efficacy is incomplete, and the spectre of resistance looms (Buxser, 2021; Van den Poel et al., 2022). Future research must focus on novel decolonization agents, perhaps leveraging probiotics, bacteriophages, or targeted antimicrobial peptides. Furthermore, we need large-scale clinical trials to determine when decolonization is most beneficial, for which patient populations, and what endpoints should be used to define success. Understanding the factors that contribute to persistent colonization, such as biofilm formation on skin and the interaction with the skin microbiome, will be essential for developing more effective strategies.

What are the precise drivers of transmission and virulence? While we understand the general modes of transmission, the specific molecular and environmental factors that drive virulence and allow *C. auris* to outcompete other skin commensals are not fully elucidated (Gómez-Gaviria et al., 2023). How does it evade the host immune system so effectively on the skin? What are the key virulence factors beyond its thermotolerance and biofilm formation? A deeper understanding of its pathogenic mechanisms, including the role of secreted enzymes like proteinases and lipases, could reveal novel targets for therapeutics that disarm the pathogen rather than kill it, potentially reducing selective pressure for resistance. Recent research has shown that *C. auris* induces pathogenic Th1 immune responses that are regulated by IL-10, allowing for long-term skin colonization (Balakumar et al., 2024; Das et al., 2025). Targeting these host-pathogen interactions may offer new avenues for preventing colonization and transmission.

How can we overcome multidrug and pan-drug resistance? The emergence of PDR strains is a critical clinical challenge. The pipeline for new antifungal agents remains limited, although promising candidates like rezafungin, ibrexafungerp, and fosmanogepix are advancing. Future strategies must go beyond traditional antifungals. Furthermore, understanding the mechanisms of antifungal tolerance—whereby fungi survive transient exposure to high drug concentrations—is crucial for preventing treatment failure (Van Rhijn and White, 2025). Targeting epigenetic regulators like the histone acetyltransferase Gcn5, which modulates ergosterol biosynthesis and drug efflux, to resensitize resistant strains to existing drugs is a novel and exciting avenue (Zhang et al., 2025b). Combination therapies, phage therapy, and immunotherapies are other areas that warrant urgent investigation.

Can we develop truly rapid, point-of-care diagnostics? While MALDI-TOF MS and PCR have improved diagnostic accuracy, they are largely confined to centralized laboratories, leading to delays in identification (Kardjadj, 2025). The development of a simple, rapid, and affordable point-of-care test suitable for near-patient use—for both colonization screening and diagnosing invasive infection—would be a major advance for infection control (Lisby and Schneider, 2021). Emerging isothermal amplification methods, such as recombinase-aided amplification (RAA) often integrated with CRISPR-Cas systems, alongside fully automated cartridge-based platforms, demonstrate significant potential for near-patient molecular diagnostics, though extensive clinical validation and scaling remain necessary (Guo et al., 2025). Such tests would enable real-time decision-making at the bedside, allowing for immediate implementation of isolation precautions and targeted therapy, potentially preventing outbreaks before they can establish.

What is the full extent of the environmental reservoir? *C. auris* has been found in diverse environments, from salt marshes to wastewater, but its complete ecological niche remains unknown. Understanding its environmental reservoirs outside of healthcare settings is crucial. Is there a significant animal reservoir? How does environmental prevalence, potentially influenced by climate change, contribute to the emergence of new clades or the introduction of strains into human populations (Jara et al., 2025)? A “One Health” approach, integrating human, animal, and environmental surveillance, is needed to answer these questions and develop comprehensive public health strategies. The discovery of *C. auris* in natural environments raises the possibility that healthcare-associated strains may originate from environmental sources, and that environmental contamination from healthcare facilities may contribute to its persistence and spread in the broader ecosystem.

## 7 Conclusion

Candidozyma auris represents a paradigm of modern multidrug-resistant pathogens, challenging healthcare systems globally. Its unique biological resilience, coupled with its ability to exploit vulnerabilities in infection control practices across diverse clinical settings, demands a more sophisticated and adaptable response. The scenario-based framework and the Integrated Control Bundle proposed in this review offer a structured, actionable paradigm to deconstruct this complex challenge. By shifting from a ‘one-size-fits-all’ mentality to tailored, evidence-based strategies specific to the ICU, LTCF, surgical, and outpatient environments, healthcare systems can mount a more effective and resource-efficient defense. Ultimate success will hinge on the synergistic application of patient-centered measures, robust facility-level environmental controls, and coordinated system-level surveillance and communication. The fight against *C. auris* is a marathon requiring sustained investment in research to close critical knowledge gaps, innovation in diagnostics and therapeutics to stay ahead of resistance, and an unwavering commitment to the fundamentals of infection prevention. By adopting a more strategic, scenario-driven approach, the global healthcare community can better protect vulnerable patients and mitigate the threat posed by this formidable pathogen.

The clinical scenario-based framework for the prevention and management of *C. auris* infections proposed herein is situated within, and serves to operationalize, the broader public health measures advocated for pathogens on the WHO Fungal Priority Pathogens List (Wang et al., 2025). Whereas overarching efforts necessarily encompass multifaceted domains—including environmental control and sanitation, surveillance and early warning systems, antifungal stewardship, and vaccine development—our framework translates these high-level objectives into a granular, ward-specific blueprint tailored to the hospital setting. In particular, it addresses a critical gap identified in the current global response: the need for effective guidelines to reduce hospital and community acquired fungal infections (Wang et al., 2025). By stratifying interventions according to distinct clinical wards—each characterized by unique patient demographics, immunological vulnerabilities, environmental pressures, and transmission dynamics—our approach provides a practical model for implementing targeted surveillance and localized infection control, both of which constitute essential pillars of a comprehensive fungal infection prevention program. Accordingly, this ward-specific strategy may serve as a translational bridge between macro-level public health imperatives and the operational realities of frontline clinical practice, offering a scalable model for mitigating the institutional burden of *C. auris* and other priority fungal pathogens.
